# Extending Palliative Approaches to Care Beyond the Mainstream Health Care System: An Evaluation of a Small Mobile Palliative Care Team in Calgary, Alberta, Canada

**DOI:** 10.1089/pmr.2021.0059

**Published:** 2022-06-01

**Authors:** Courtney Petruik, Simon Colgan

**Affiliations:** ^1^ Department of Sociology, University of Calgary, Calgary, Alberta, Canada.; ^2^ Calgary Allied Mobile Palliative Program (CAMPP), Calgary, Alberta, Canada.

**Keywords:** community-based palliative care, end-of-life care, evaluation, palliative care, social marginality

## Abstract

**Background:**

People experiencing houselessness (PH) endure worse health outcomes than their housed counterparts and often have inadequate care when nearing end of life. Innovative palliative care approaches are necessary when considering socially vulnerable populations.

**Aim:**

Evaluate the implementation and early outcomes of the Calgary Allied Mobile Palliative Program (CAMPP) after the first four years of servicing people experiencing extreme social marginality.

**Setting/Participants:**

Participants include CAMPP clients and service providers (SPs) who work adjacently to CAMPP in the social services/health sectors.

**Design:**

This is a mixed-methods evaluation, including an SP survey (*n* = 31); client interviews (*n* = 5); collection of program metrics; and case note reviews.

**Results:**

The CAMPP has served 128 clients to date. The CAMPP supported clients by connecting them to 62 services, programs, agencies, and/or resources totaling 485 connections. The most referred-to resource was for social support in the community for PH at 61 referrals. The second was for transportation with 57 referrals, followed by referrals to palliative and Home Care programs (*n* = 53 referrals). Another common referral was for food assistance with 30 referrals. The survey showed that 97% of SPs agreed that CAMPP is “an essential service in the area of palliative care.” Twenty-six of 30 (87%) “Strongly Agreed” or “Agreed” that their knowledge in working with people with life-limiting illnesses has improved since working with CAMPP. The SPs suggested that the team should focus on referral clarity and improved communication with the wider health care team. Finally, clients reported high levels of satisfaction with CAMPP services. Clients also reported challenges navigating the complexity of care in the social/health services sector.

**Conclusions:**

The CAMPP bridges the gap in care between health/social services. The CAMPP connects clients to community resources and is effective in adapting to client needs. This evaluation provides four recommendations to improve and build on the existing program.

## Background

More than 235,000 Canadians experience houselessness annually.^1^ Health outcomes for persons experiencing houselessness (PH) are worse than their housed counterparts and can lead to premature death, with unique needs at the end of life.^1–3^ “Double vulnerability” occurs when a person suffers the negative consequences from the social/health vulnerabilities of houselessness^3^ and are at a greater risk of serious illnesses such as cancer, cardiovascular disease, respiratory illness, infectious disease, and injury.^2^ Further, life expectancy is reduced for PH with a mean age at death between 34 and 47 years, which is drastically lower than the 81.95 years that the general population expects.^4^

In O'Connell's literature review on premature mortality in houseless populations, he explained that medical care for PH is challenging within our mainstream health care model. He noted that PH had to focus on the immediacy of their daily struggle for safe shelter and meals, pushing health needs down the priority list. He stated that for PH, common ailments often progressed untreated, leading to increased numbers of emergency department visits and hospital admissions.^3^

Many illnesses and premature deaths are preventable through the provision of stable housing and access to proper health care.^2,4^ However, until long-term societal-level solutions are implemented, houselessness and health care barriers for PH remain social issues. In the meantime, health/social sector workers have responded with not-for-profit grassroots programs aiming at mitigating these hardships and preventing poorer health outcomes for PH.

### The development of Calgary Allied Mobile Palliative Program

In 2016, Dr. Simon Colgan and colleagues developed the Calgary Allied Mobile Palliative Program (CAMPP) to address the needs of PH with life-limiting illnesses struggling to receive adequate care by the health system. Before CAMPP, no such palliative services existed in Calgary that focused solely on supporting PH.

The CAMPP is funded by several philanthropic organizations and comprises a nurse coordinator, a health navigator, and the weekly support of a palliative physician who is funded by an “Alternative Relationship Plan” that supports Albertan physicians who participate in programs but do not charge a fee for service. The CAMPP provides outreach support, monitoring of health/social situations; palliative consultations; connections to community resources; housing support; banking and daily activity assistance; medication education; advocacy; therapeutic listening; grief support; and advanced care planning. A typical CAMPP client is nearing end of life; however, for some clients who access the program, “graduation^*^” is possible once they become well supported.

This evaluation outlines program activities and guides program development with the intention of improving program sustainability and informing future palliative equity practices.

## Methods

This evaluation adhered to utilization-focused evaluation values,^5^ ensuring that evaluation activities were fiscally and temporally feasible. The project team completed the Alberta Research Ethics Community Consensus Initiative (ARECCI) screening tool designed to help project leads mitigate ethical risks by providing decision-support tools, training opportunities, and project ethics consultation.^6^ The screening tool scored this project as “minimal risk,” therefore the team did not pursue ARECCI's Second Opinion Review.

Given that clients were individuals made vulnerable by physical and social circumstances and relied on CAMPP's care, we informed the individuals that their care was not affected by participating/not participating. We ensured that participation was voluntary and informed by reviewing the consent and information forms and addressing questions. We allowed interviewees to choose time/place of the interviews. Safety and comfort were important ethical considerations and corresponded to our responsibility to protect vulnerable participants. We informed participants of anonymity and confidentiality risks, and we removed all identifying information.

### Health and social service providers survey

The survey was sent to 53 service providers (SPs). The SPs were those providing social and/or health care to the PH in Calgary, Canada. The survey yielded 31 responses. This 58% response rate is acceptable for online surveys in program evaluations.^7^ The survey comprised 10 questions. According to the ethical guidelines of utilization-focused evaluations^8^ recommending that evaluators capture the minimum data required, the survey was kept brief. The survey was hosted via *SurveyMonkey*, and data were exported via an excel spreadsheet and analyzed manually.

### Client interviews

Clients were individuals referred to CAMPP via the program's referral process. Referrals start with a faxed/emailed referral form into the program. The referrer can also contact CAMPP via phone, text, or e-mail. The criteria for someone to become a CAMPP client is houselessness/vulnerable housing and life-limiting illness. The CAMPP asked clients whether they consented to be contacted and if they agreed, an evaluator phoned to schedule an interview.

Five clients were interviewed. The interviewees worked with CAMPP for weeks to years and were 37–66 years of age. Three participants identified as male and two as female. The interviews were audio-recorded, lasted 15–30 minutes, and followed a semi-structured interview guide. Participants were provided $15.00 for their time.

### Program metrics and case note reviews

The case note reviews explored the number and type of CAMPP-initiated connections with resources, agencies, programs, and services. The CAMPP provided program metrics via internally tracked program outcomes, which were shared aggregately with the evaluators. Program metrics reflected work that took place between October 1, 2016 and July 8, 2020. The evaluation team reviewed 128 case notes tallying the referrals to external care providers, services, programs, agencies, and resources.

Aggregated data tracking referrals to income supports, housing, medication coverage, and primary care physicians (PCPs) were also provided via internally tracked program outcomes.

### Analytical approach

Evaluators analyzed qualitative data by using content-analysis, a research technique for the systematic description of content using themes. Patterns within the data were sorted and categorized into themes based on their repetition and stated importance of the topic by participant(s).

## Results

### Program metrics

Of the 128 clients whom CAMPP served, 29 (23%) identified as female and 99 (77%) as male. No non-binary clients participated. The average client was 58.9 years. Clients ranged from 22 to 86 years. The most common primary diagnosis for CAMPP clients was cancer. Twenty-three (18%) clients were referred from CAMPP's partner agency, CUPS Calgary Society.^†^ Acute Care Hospital Units referred 47 people (37%), and other community partners referred 58 (45%).

Thirty-seven people (29%) presented to CAMPP without a PCP. Of this 29%, 60% (*n* = 22) connected with a PCP after accessing CAMPP. In addition, 63 clients were unhoused at time of referral. Of these 63 clients, 51% were housed since CAMPP involvement; however, this number did not account for changing residences occurring due to housing loss or voluntary moves.

Similarly, of the 23 clients who had no source of income at time of referral to CAMPP, 12 (52%) were connected to publicly provided income supports (i.e., Alberta Works Income Support, Alberta Income for the Severely Handicapped, Canada Pension Plan, and Old Age Security) after involvement with CAMPP.

Twenty-one individuals presented to CAMPP without medication coverage. After connecting with CAMPP, 13 secured coverage (62%). There were 33 (26%) direct Home Care referrals from CAMPP and 51 (40%) clients who completed Advanced Care Plans with designated Goals of Care (Table 1).^‡^

**Table 1.** Client Characteristics Summary Table

Client characteristic	Count	Total clients
Gender distribution	Males = 99 (77%) Females = 29 (23%)	*n* = 128
Age range	22–86 Years	*n* = 128
Most common diagnosis	Cancer	*n* = 128
Most common referral source	Calgary acute care hospital units	*n* = 128
Connected to a PCP	*n* = 22 (60% of those presenting without a PCP on intake)	*n* = 37
Connected to housing	*n* = 32 (51% of those presenting without housing on intake)	*n* = 63
Connected to source of income	*n* = 12 (52% of those presenting without a source of income on intake)	*n* = 23
Secured medication coverage	*n* = 13 (62% of those presenting without medication coverage on intake)	*n* = 21
Completed advance care plans with designated goals of care	*n* = 51 (40%)	*n* = 128

PCP, primary care physician.

### Client case note reviews

The CAMPP connected clients to 62 services, programs, agencies, and/or resources totaling 485 connections. Table 2 lists resources that have common services under one heading (e.g., legal support). Therefore, the list does not show all 62 services, but rather a categorized list totaling 45 services. All connections to resources were CAMPP-initiated and excluded connections by other SPs during involvement with CAMPP (e.g., a referral made by a PCP to a hepatology clinic).

**Table 2.** Calgary Allied Mobile Palliative Program Connected Services Summary Table

Service	No. of referrals
Addictions Recovery Centers	10
Access Calgary (public transportation)	24
Accessible transport/taxi vouchers	33
AHS Capacity Assessments	6
AHS Cardiac Function Clinic	3
AHS Community Paramedics	6
AHS Dialysis	2
AHS Dysphasia	1
AHS Ethicist	1
AHS Hepatology	1
AHS Indigenous Patient Navigator	8
AHS Long-Term Care	8
AHS MAiD	11
AHS Occupational Therapy	7
AHS Palliative Consultation	21
AHS Palliative or Integrated Home Care	53
AHS Patient Relations	1
AHS Paracentesis	2
AHS Spiritual Care or Chaplain	8
AHS STI Clinic	1
AHS Wound Care Clinic	1
Banking Support	19
CCAIL	3
Community Rehabilitation	1
CUPS	24
Dental	5
Developmental Disability Support	1
Documentation Support (Identification, Death Certificate, Social Insurance Number, Alberta Health Card)	11
Faith-based Community Non-Profits	2
Food assistance	30
Give a Mile (Support for air transport for loved ones of people in palliative care)	5
Grief and Estate Planning	6
Hospice	20
Household and Personal Supplies	21
Legal Supports	9
Medical Examiner	1
Medical supplies	36
Multiple Sclerosis Society	3
Optometry/Ophthalmology	1
Psychologist	4
Physiotherapist	1
Seniors' Support (65 years+)	11
Sleep Clinic	1
Social support programs for houselessness	61
Speech and Language Pathologist	1
Total	485

AHS, Alberta Health Services; CCAIL, Calgary Aids to Daily Living; MAiD, Medical Assistance in Dying.

The most referred-to resource was for “social supports in the community” at 61 referrals. The second was “transportation” with 57 referrals. This was followed by referrals to “Palliative and Integrated Home Care” (*n* = 53 referrals). Another common referral was for “medical supplies” and “food assistance” with 36 and 30 referrals, respectively.

The CAMPP referred to their partner agency (CUPS) 24 times. Notably, 21 referrals were made for palliative consults and 20 to hospices. The CAMPP facilitated 11 connections to the Medical Assistance in Dying (MAiD) program.^§^ The number of connections to MAiD did not mean clients died by MAiD, rather it reflected clients who were connected to MAiD for services and/or for information gathering.

### SP survey

The SPs reported moderate familiarity (average rating = 6.96/10) with palliative approaches to care for PH and moderate comfort in identifying clients who required palliative care (average rating = 7.32). Twenty-six of the 30 (87%) respondents “Strongly Agreed” or “Agreed” that their knowledge in working with people with life-limiting illnesses improved since working with CAMPP. Four respondents (14%) “Neither Agreed nor Disagreed” to the same question (Table 3).

**Table 3.** Summary Table of Service Provider Responses

Questions		Results
**Category: Current knowledge**		
Question 1	Rating Scale Question	Responses (*n* = 31)
To what extent are you familiar with palliative approaches to care for the population you work with?	(1 = very little familiarity and 10 = very familiar)	Rating of 10 (*n* = 6) Rating of 9 (*n* = 3) Rating of 8 (*n* = 4) Rating of 7 (*n* = 4) Rating of 6 (*n* = 3) Rating of 5 (*n* = 8) Rating of 4 (*n* = 2) Rating of 3 (*n* = 1) Rating of 2 (*n* = 0) Rating of 1 (*n* = 0)
Question 2	Rating Scale Question	Responses (*n* = 31)
To what extent do you feel comfortable identifying clients who may require palliative care?	(1 = not comfortable at all and 10 = very comfortable)	Rating of 10 (*n* = 8) Rating of 9 (*n* = 3) Rating of 8 (*n* = 4) Rating of 7 (*n* = 4) Rating of 6 (*n* = 4) Rating of 5 (*n* = 5) Rating of 4 (*n* = 2) Rating of 3 (*n* = 1) Rating of 2 (*n* = 0) Rating of 1 (*n* = 0)

**Category: Education, Advocacy, and Capacity Building**		
Question 3	Rating Scale Question	Responses (*n* = 31)
To what extent do you feel CAMPP is an essential SP of people experiencing homelessness?	(1 = not essential at all and 10 = extremely essential)	Rating of 10 (*n* = 25) Rating of 9 (*n* = 2) Rating of 8 (*n* = 3) Rating of 7 (*n* = 0) Rating of 6 (*n* = 0) Rating of 5 (*n* = 0) Rating of 4 (*n* = 0) Rating of 3 (*n* = 1) Rating of 2 (*n* = 0) Rating of 1 (*n* = 0)
Question 4	Likert Scale Question	Responses (*n* = 30)
My knowledge about working with people with life-limiting illnesses has improved since collaborating alongside CAMPP	(Strongly Disagree [SD], Disagree [D], Neither Disagree nor Agree [ND/A]; Agree [A]; Strongly Agree [SA])	SA = 13; A = 13; NAD = 4; SD = 0; D = 0
Question 5	Choice Question	Responses (*n* = 30)
I have attended a presentation, workshop, or seminar held by one or more members of CAMPP regarding their areas of expertise (palliative approaches to care and/or homelessness)	(Yes, No, Does not Recall)	Yes = 17; No = 11 Does not recall = 2
Question 6	Likert Scale Question	Responses (*n* = 30)
I would be interested in attending a presentation, seminar, or workshop held by CAMPP focusing on palliative approaches to care for people experiencing homelessness	(Strongly Disagree [SD], Disagree [D], Neither Disagree nor Agree [ND/A]; Agree [A]; Strongly Agree [SA])	SA = 22; A = 6; NAD = 1; SD = 0; D = 1
Question 7	Likert Scale Question	Responses (*n* = 30)
Since working with CAMPP, I feel better prepared when confronted with possible palliative cases in the client population.	(Strongly Disagree [SD], Disagree [D], Neither Disagree nor Agree [ND/A]; Agree [A]; Strongly Agree [SA])	SA = 15; A = 12; NAD = 3; SD = 0 D = 0
Question 8	Likert Scale Question	Responses (*n* = 30)
When necessary, I feel comfortable connecting with CAMPP for assistance regarding a client who may be suitable for palliative care	(Strongly Disagree [SD], Disagree [D], Neither Disagree nor Agree [ND/A]; Agree [A]; Strongly Agree [SA])	SA = 23; A = 6; NAD = 1; SD = 0; D = 0

**Category: Closing**		
Question 9	Likert Scale Question	Responses(*n* = 30)
I have felt or would feel comfortable referring a client to CAMPP.	(Strongly Disagree [SD]; Disagree [D]; Neither Disagree nor Agree [ND/A]; Agree [A]; Strongly Agree [SA])	SA = 24; A = 6; NAD = 0; SD = 0; D = 0
Question 10	Qualitative/Open Ended	*N* = 22
Please share what you feel CAMPP has done well and/or any suggestions or feedback you would like to offer for program improvement.		Themes listed in text.

CAMPP, Calgary Allied Mobile Palliative Program; NAD, Neither Agree or Disagree; SP, service provider.

Thirty respondents reported >8 out of 10 when asked, “To what extent do you feel CAMPP is an essential SP of palliative approaches to care for persons experiencing homelessness?” A one rating meant “not essential at all” and 10 meant “extremely essential.” One respondent chose three; the average rating was 9.5.

The survey asked whether respondents had attended or would be interested in attending a presentation/workshop/seminar by CAMPP on palliative approaches to care and homelessness. Twenty-eight respondents of the 30 (93%) reported interest, with one respondent (3%) disagreeing and one respondent (3%) neither agreeing nor disagreeing with the statement.

Twenty-seven respondents (90%) reported “Strongly Agreeing” or “Agreeing” that since working with CAMPP, they were better prepared when confronted with possible palliative cases. Thirteen percent (*n* = 4) neither “Agreed” nor “Disagreed” with this statement. When participants were asked whether they felt comfortable connecting with CAMPP for assistance with a client who may be suitable for palliative care, everyone “Agreed” or “Strongly Agreed” except one respondent who reported that they “Neither Agreed nor Disagreed.” Participants responded affirmatively when asked whether they were comfortable referring someone to CAMPP, with everybody “Strongly Agreeing” (*n* = 24) or “Agreeing” (*n* = 6).

The open-ended question was completed by 22 SPs. Responses were categorized into two themes, “CAMPP is a Needed Service” and “Suggestions for the Future.”

#### Theme 1: CAMPP is a needed service

Seven SPs (32%) reported that CAMPP was distinctive in their high responsiveness and accessibility. They also described CAMPP as a “low-barrier” program that is helpful for PH.

[CAMPP] is extremely low barrier programming available to our most vulnerable folks.—SP

The SPs viewed CAMPP as effective due to their meaningful relationship-building with colleagues and clients, accepting and compassionate perspectives, and non-judgmental approach (*n* = 10; 45%).

CAMPP is a dependable, compassionate, and responsive team to collaborate with. We have a number of individuals in our buildings that were referrals from CAMPP and our teams have developed a strong partnership to ensure appropriate and compassionate care.—SP

Respondents (*n* = 10; 45%) noted that CAMPP's ability to bridge gaps in care were often due to advocacy efforts and excellent communication.

[CAMPP] has great communication with all aspects of the care team and has acted as a communication liaison between myself and other team members and the patient when the patient is difficult to reach by phone. CAMPP has helped patients with housing, transportation, and more and have gone above and beyond for patients to ensure their last months are optimized and spent in the home where the client wishes to be.—SP

The SPs (*n* = 8; 36%) stated that CAMPP staff were knowledge experts, educators, and advisors in palliative care and houselessness.

CAMPP has been essential to the education and care delivery in hospices and ongoing collaboration with CAMPP is needed to continue to build in [end of life care].—SP

Survey respondents affirmed the work of the CAMPP team in the areas cited earlier; however, there were also suggestions for future consideration.

#### Theme 2: Suggestions for the Future

Participants reported the need for streamlined communication, including clarity around identifying the attending PCP.

…what hasn't gone as well [with CAMPP] is ensuring that everyone on the care team knows who the PCP is [for the client] and that all discharge summaries, consultation notes, lab tests, and oncologist notes were CC'd to the PCP.—SP

In addition, one SP faced uncertainty in identifying appropriate clients to refer to CAMPP.

My only feedback is to make it easier for frontline staff to be able to assess clients if they are palliative or not. Maybe look at creating a system where it is not the responsibility of frontline staff to recognize and make referrals.—SP

Suggestions for the future focused on referral clarity and improving communication with the wider health care team.

### Client interviews

Clients assessed CAMPP positively, stating that CAMPP was invaluable, stabilizing, and improved their well-being.

The people that work [at CAMPP] are phenomenal. The help they have given me has been invaluable. Its like a one-stop-shop; they are easily accessible, so I just make a call and they help me find the answers. They are “my peoples.” I will work with them until I am, you know, done.—Client

Clients found CAMPP useful in helping with daily activities and basic needs such as transportation to medical appointments, access to food, and managing appointments and medication schedules.

If I had an appointment that was hard for me to get to, [CAMPP] would write out a taxi voucher for me so I could get to these appointments and not worry. An appointment may be only three blocks away but for me to get there is murder because my kidneys don't function and I just tire out. Helping with my appointments makes life easier.—Client

Clients stated that underpinning CAMPP's service was a non-judgmental and friendly approach that helped build relationships.

They helped me stay on top of things and my situation in a positive way. I never felt judged even if I didn't do everything right.—Client

Some clients were unsure of who to contact since they had “too many workers.”

I don't know what they [CAMPP] do. I don't know who does what. I don't really care as long as something gets done.—Client

Overall, clients were satisfied and assessed CAMPP positively, noting suggestions for improvement regarding clarity of SP responsibilities.

### Recommendations

The evaluation team offered recommendations that came from the evaluation findings. Recommendations included three actions that the team could “start to” implement and one that they should “continue” (Table 4).

**Table 4.** List of Evaluation Recommendations

Areas	Description	Recommendation
Start to…		
1. Improve clarification and education around palliative suitability of potential clients to adjacent SPs from the health and social sectors	To provide education to adjacent SPs around identifying palliative care needs among their clients and how to identify an appropriate referral to the program.	(1) The development and provision of preliminary client palliative assessments available to adjacent SPs; and/or clarified referral criteria listed on a referral form and on other public facing content (e.g., website, brochures, etc.) (2) Ongoing availability of educational materials such as delivery of workshops to potential referrers in both health and social sectors. Including the addition of a “lived experience facilitator” (i.e., a person who has had similar experiences as CAMPP clients or a CAMPP client themselves) in workshops could also strengthen the teachings. (e.g., through the medium of online workshops and/or educational documentation for dissemination.)
2. Improve clarification of program activities and responsibilities amid the wider milieu of health and social services	To ensure all potential referrers, health care team members, adjacent health care portfolios, and clients are clear on the services provided by CAMPP Minimize confusion around primary clinical responsibility of clients to help reduce overlap in services and/or mistakes in service provision	(1) Schedule a strategic planning session for the team and necessary stakeholders, including representatives from both health and social services (e.g., From Alberta Health Services and/or a Homeless Serving Sector System Planner from Calgary Homeless Foundation) to help bring consensus regarding organizational priorities among key stakeholders and to help the program clarify program aims and activities (2) Continue educational activities that the program is already engaging in, such as program presentations, to community stakeholders (3) Improve online presence through the development of an updated website and/or social media accounts to help inform community members about recent and upcoming program happenings
3. Implement ongoing program monitoring and evaluation activities as well as establish an agreed-upon data-sharing system across health and social sectors	To understand whether CAMPP activities continue to be effective and to incorporate a mechanism to track program adaptations and their implementation To ensure that relevant program data from influential and relevant organizations such as Alberta Health Services and Calgary Homeless Foundation are accessible for meaningful reporting on program outcomes	(1) Improved monitoring, and evaluation activities including clarified processes for ongoing data collection metrics (2) Use of a framework for organizing evaluation activities (e.g., The use of the RE-AIM model. The RE-AIM is a widely used evaluation framework for health and social services that has previously been utilized in projects on vulnerable populations such as older adults^12^ as well as in many other practice areas). (3) Develop a working group focusing on establishing a data-sharing plan and agreement with relevant parties such as AHS and CHF.
Continue to…		
4. Support clients and support adjacent SPs with navigating the complex system of health and social services	To continue excellent navigation of the health and social needs of PEH with life-limiting illnesses and to ensure clients obtain appropriate care to maximize well-being and minimize discomfort	(1) Continue mobile outreach efforts to support clients in the community to help reduce access barriers to health and social services (2) Continue supporting clients by using evidence-based best practices using a non-judgmental, harm-reduction, individualized, and relationship-centered approach (3) Continue supporting clients in navigating the health system, including improving access to services such as housing, income supports, primary care, legal supports, health services, and completion of advance care plans

## Conclusions

The CAMPP has taken steps to improve system navigation by advocating for change by promoting health equity in palliative care as a core program tenet. Further, non-judgmental, and relationship-centered support (i.e., harm-reduction focused and trauma-informed) is critical for clients and CAMPP excelled in this area.

The CAMPP improves care for people living with life-limiting conditions who experience complex service needs and has reduced barriers to services as demonstrated by their extensive work connecting clients to resources, programs, agencies, and services across health/social sectors. The CAMPP facilitates the palliative consultation process and has graduated clients who no longer required palliative support. The CAMPP also connects clients to existing social/health services and delivers education and capacity-building for SPs in palliative care and houselessness demonstrating an effective and essential cross-sectoral approach to their work.

The CAMPP demonstrates that they bridge gaps in care for clients. The SPs and clients describe CAMPP as integral to caring for PH because of their palliative care model. If the program sustains long-term funding and if it remains intact in its current structure and delivery, the evaluation findings affirm that the program would complement the broader mainstream health system by servicing socially vulnerable people.

## Discussion

This evaluation took a mixed-methods approach, including an SPs survey, program metrics, and qualitative interviews. The web-based and brief survey attempted to maximize completion rates and minimize the attrition of busy SPs, as shown by the 96% completion rate; this was higher than the average online survey completion rate of 87%.^7^

The CAMPP connected clients to services, resources, programs, and agencies 485 times during the data collection period. These connections are crucial for PH with life-limiting illness since traditional models of palliative care are largely inaccessible to them.^9^ In Canada's universal health system, universality does not ensure health equity^3^ and the CAMPP team addresses this by providing community-based care and liaising with other SPs.^3^

The CAMPP provides a palliative approach that is beneficial to clients and reduces inequities in access. The CAMPP's multi-faceted, intentional, harm-reduction, and trauma-informed approach is cited in the literature as crucial in the community of PH with life-limiting illnesses and is often missed in mainstream health systems.^1^ Socially vulnerable individuals are often unable to access health care, including medication coverage and/or access to affordable, appropriate housing.^9,10^ The CAMPP addresses these gaps, providing relevant and important care as evident in the findings.

### Limitations

This evaluation had several limitations. First, the program data were not intended for evaluation; therefore, they did not include all CAMPP's activities. Second, CAMPP requested permission for evaluators to contact clients for interviews and of those agreeing to be contacted, most did not have reliable telephone access, which impeded their ability to participate. This likely reduced the diversity of responses. Lastly, comparison data and data from Alberta Health Services (AHS) were inaccessible, which would have provided an enhanced understanding of CAMPP's impact on system and health outcomes.

## Abbreviations Used

CAMPP: Calgary Allied Mobile Palliative Program
MAiD: Medical Assistance in Dying
PCP: primary care physician
PH: persons experiencing houselessness
SP: service provider

## Notes

^*^
CAMPP “graduates” client if they become well supported in the community and no longer require intensive CAMPP support. The team does not take statistics on the number of graduates, as this term refers to an informal process of “stepping back” from working closely with the client. The CAMPP and the client have ongoing discussions about the level of care provided and decide together whether the client should be “graduated” or not and this status may change at any point based on their level of need.

^†^
CUPS Calgary Society is a charitable organization located in Calgary, Alberta, Canada that assists adults and families in Calgary living with the adversity of poverty and traumatic events to become self-sufficient. Note: “CUPS” is not an abbreviation but the name of the organization.

^‡^
Advance Care Planning is a process used in Alberta, Canada as a way to help you think about, talk about, and document wishes for health care in the event that you become incapable of consenting to or refusing treatment or other care. Goals of Care Designation is a medical order used in Alberta, Canada to describe and communicate the general aim or focus of care, including the preferred location of that care.^11^

^§^
MAiD is an option available to Albertans nearing end of life. It allows adults suffering from a grievous and irremediable medical condition to voluntarily request medicine that will bring about their death.
